# Recognition Characteristics of Facial and Bodily Expressions: Evidence From ERPs

**DOI:** 10.3389/fpsyg.2021.680959

**Published:** 2021-07-05

**Authors:** Xiaoxiao Li

**Affiliations:** Academy of Psychology and Behavior, Tianjin Normal University, Tianjin, China

**Keywords:** facial expressions, bodily expressions, event-related potential, P1, N170, N2, P3

## Abstract

In the natural environment, facial and bodily expressions influence each other. Previous research has shown that bodily expressions significantly influence the perception of facial expressions. However, little is known about the cognitive processing of facial and bodily emotional expressions and its temporal characteristics. Therefore, this study presented facial and bodily expressions, both separately and together, to examine the electrophysiological mechanism of emotional recognition using event-related potential (ERP). Participants assessed the emotions of facial and bodily expressions that varied by valence (positive/negative) and consistency (matching/non-matching emotions). The results showed that bodily expressions induced a more positive P1 component and a shortened latency, whereas facial expressions triggered a more negative N170 and prolonged latency. Among N2 and P3, N2 was more sensitive to inconsistent emotional information and P3 was more sensitive to consistent emotional information. The cognitive processing of facial and bodily expressions had distinctive integrating features, with the interaction occurring in the early stage (N170). The results of the study highlight the importance of facial and bodily expressions in the cognitive processing of emotion recognition.

## Introduction

In daily life, both the face and body can convey emotional information. For example, sad faces are often accompanied by body expressions such as lowering of the head, happy faces are accompanied by body gestures such as dancing with joy, and angry faces are accompanied by body expressions such as clenched fists and stomping (Proverbio et al., 2018). Some researchers suggest that bodily expressions are more reliable than facial expressions (Van den Stock et al., 2007) because people are often able to hide their real emotions in their faces, such as through fake smiles. Accordingly, emotional body language (EBL) refers to the integration of emotional information, coordinated meaningful movement, and behavior expressed by the body (de Gelder, 2006). Body movement and posture also convey emotion-specific information (Dael et al., 2012; Witkower and Tracy, 2019; Calbi et al., 2020). Thus, it is natural to consider how facial and bodily expression recognition interact. Numerous studies suggest that facial expression recognition is influenced by multiple contextual factors (Aviezer et al., 2012b; Van den Stock et al., 2014; Neath-Tavares and Itier, 2016; Xu et al., 2017; Zhang et al., 2018). Therefore, an increasing number of researchers are exploring bodily expressions and their congruency with facial expressions (Van den Stock et al., 2011; Downing and Peelen, 2016; Poyo Solanas et al., 2018). In this study, we used event-related potential (ERP) to explore the differences in the recognition of facial and bodily expressions, as well as the characteristics of their interaction over time.

Relevant studies provide evidence for the interaction between facial and bodily expressions. Meeren et al. (2005) first combined facial and bodily expressions to produce matched/mismatched emotional compounds for facial expression recognition. The results showed the emotional congruence effect of facial and bodily expressions (Van den Stock et al., 2007). Additionally, bodily expressions can compensate for emotional information missing from facial expressions. When using ambiguous facial expressions (high-intensity, such as lose and win), bodily expressions played a more important role than facial expressions, shaping the perceived affective valence of intense expressions (Aviezer et al., 2012a). The results of an eye movement experiment also showed that when the emotion of facial expressions was inconsistent with that of bodily expressions, the fixation pattern was affected by bodily expressions (Aviezer et al., 2008). Moreover, the emotional congruence effect has also been found in the auditory, olfactory, and audiovisual fields (Föcker et al., 2011; Hietanen and Astikainen, 2013; Leleu et al., 2015). Calbi et al. (2017) used an expression matching task, requiring the judgment of emotional congruence between sequentially presented pairs of stimuli belonging to the same category (face–face or body–body) and between stimuli belonging to different categories (face–body or body–face). The results showed a strict link between emotions and action. The evidence suggests that bodily expressions are essential in understanding emotions, but the difference in how bodily and facial expressions contribute to emotional perception requires further study.

Some researchers have attempted to study the interaction between facial and bodily expressions using ERP (a special kind of brain evoked potential that collects the fluctuation caused by nerve activity measured in milliseconds), but few have revealed its neural characteristics accurately. Here, we discuss four specific ERP components related to the perception of facial and bodily expressions: P1, N170, N2, and P3. Meeren et al. (2005) found that, like facial expressions, bodily expressions can evoke P1, a positive component of the bilateral occipital electrode caused by visual stimulation in the early stage of visual processing. Some researchers believe that P1 shows a larger amplitude to negative faces, which is believed to indicate that low-frequency spatial information is highly sensitive to negative faces. For example, Luo et al. (2010) found that bodily expressions processed in the early stages (P1) are more sensitive to threatening information. N170, a negative deflection detected in the lateral occipito-temporal electrode, can distinguish between faces and objects. Therefore, N170 is regarded as an essential component of facial configuration processing (Pegna et al., 2008; Tanaka, 2016). Although the body can induce N170, the amplitude is not as large as that of the face, and the neural basis may not be the same. Similarly, N170 is also induced in response to an inverted body with a larger amplitude than that of the face (Minnebusch and Daum, 2009). Whether different bodily expressions have a statistically significant effect on N170 needs further study (de Gelder et al., 2015). Borhani et al. (2015) found that the N170 latency of bodily expression is significantly later than that of facial expression; therefore, it has been named N190. However, there are inconsistent conclusions about whether N170 is influenced by emotions (Ashley et al., 2004; Rellecke et al., 2012). Finally, P300, also known as P3, and the subsequent slow wave (PSW) are considered late positive potential (LPP) components. P300 is a component that is mainly related to high-level processing of cognitive activities by people engaged in certain tasks, such as attention, discrimination, and working memory (Recio et al., 2017).

Gu et al. (2013) used ERP to study the interaction between facial and bodily expressions and proposed a three-stage model of processing of facial and bodily expressions. The first stage is characterized by automatic and rapid extraction of threatening information from bodily expressions; the second stage detects any inconsistent information between facial and bodily expressions; and the third stage entails finer processing integration and judgment. Regarding the first stage, Poyo Solanas et al. (2020) posited that body posture not only sends emotional information to us but also provides motion information. Overall, bodily and facial expressions have been found to overlap in neural processing mechanisms, and there may be interactions between them (Zhu and Luo, 2012; Hietanen et al., 2014; Borgomaneri et al., 2015; Borhani et al., 2016). However, Liang et al. (2019) used fMRI to explore the network representation of facial and bodily expressions and found that the human brain employs separate network representations for facial and bodily expressions of the same emotions. In this study, we expand on the two emotions of happiness and fear examined by Gu et al. (2013) to explore whether the identification of positive and negative emotions is consistent with the three-stage model and the integration characteristics of facial and bodily expressions.

Although some studies have begun to explore the interaction between bodily and facial expressions, they focus mostly on understanding the behavioral results of this interaction and the characteristics of the early components of ERP. There are some consistencies and differences between the cognitive processing mechanisms of bodily and facial perception. Therefore, this study intends to explore the temporal characteristics of cognitive processing of facial and bodily expressions using ERP. The experimental task was manipulated to allow us to explore the interaction between facial and bodily expressions by asking participants to judge the facial and bodily expressions separately and together for both consistent and inconsistent emotional expressions.

## Materials and Methods

### Participants

A total of 33 (16 males) right-handed undergraduates ranging from 18 to 26 years participated in the study. Five participants were excluded: four due to equipment problems, and one for reversing the task instructions. Thus, there were 28 effective participants. All participants had a normal or a corrected-to-normal vision. All participants volunteered to participate in the experiment and provided written informed consent.

### Materials

Images of facial and bodily expressions were taken from two image databases: the Chinese Affective Picture System (CAPS) and the Bochum Emotional Stimulus Set (BESST) picture library. All the body pictures in the BESST picture library were covered with faces, the background of all pictures was gray, and all limbs appeared in the middle of the pictures. Eighty images were selected, half of which were male and female, and half were positive and negative.

Ten psychological postgraduates scored the valence and arousal of 80 face pictures and 80 body pictures using a 9-point Likert scale. Valence refers to the degree of pleasure expressed by the picture itself, from very pleasant to very unpleasant. Arousal refers to the degree of arousal of the emotion, from calm to excitement. There was no time limit for stimuli presentation or response from participants. The average score for each picture was taken as the average score of the 10 participants. Finally, 60 pictures each of effective facial and bodily expressions were selected. Positive pictures only included happiness, while negative pictures included anger, disgust, fear, sadness, and surprise. ANOVA showed that the valence of negative (2.958 ± 1.014) facial expression pictures was significantly lower than that of positive (6.223 ± 1.042) facial expression pictures [*F*(1, 9) = 72.01, *p* < 0.001, η*^2^* = 0.889] and the valence of negative (2.893 ± 0.803) bodily expression pictures was significantly lower than positive (6.537 ± 1.384) bodily expression pictures [*F*(1, 9) = 34.33, *P* < 0.001, η*^2^* = 0.792]. There was no significant difference between the arousal degree of negative facial expression pictures (4.840 ± 1.904) and positive facial expression pictures (4.490 ± 1.691) [*F*(1, 9) = 0.40, *p* > 0.05] or between the arousal degree of negative bodily expression pictures (4.657 ± 1.840) and positive bodily expression pictures (5.290 ± 1.901) [*F*(1, 9) = 1.72, *p* > 0.05].

**FIGURE 1:**
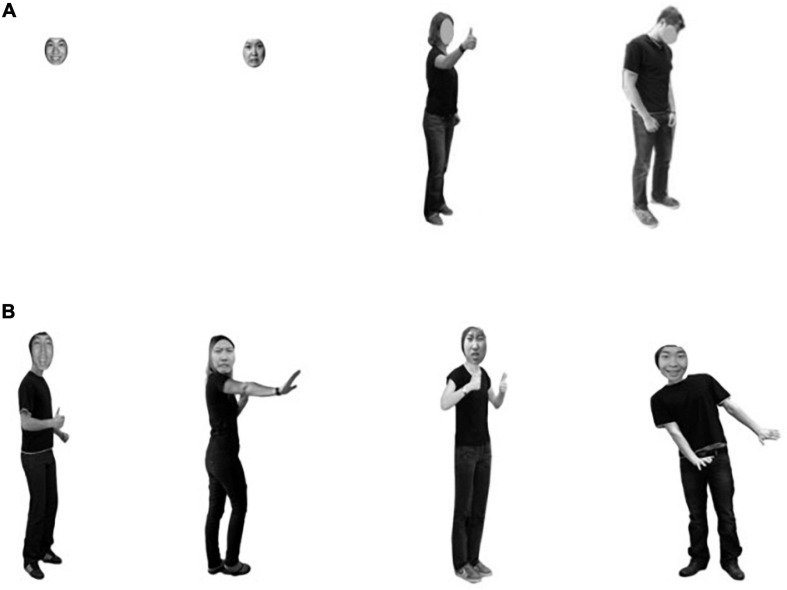
Schematic diagram of experimental materials. **(A)** The pictures representing emotions separately for face and body presented are positive-face, negative-face, positive-body, and negative-body. **(B)** Four kinds of facial and bodily compound stimuli were produced in the experiment. From left to right, they are positive face–positive body, negative face–negative body, positive face–negative body, and negative face–positive body. (All experimental materials were edited with Adobe Photoshop CS6 to control the brightness and contrast).

Adobe Photoshop CS6 was used to combine facial and bodily expressions. Two kinds of facial expressions and two kinds of bodily expressions were combined to produce four kinds of face–body combination stimuli: positive face–positive body, negative face–negative body, positive face–negative body, and negative face–positive body. Among them, the first two were expression consistent combinations, and the latter two were expression inconsistent, each with 15 pictures, for a total of 60. The contrast and luminance of face–body combination stimulation were controlled. The face-to-body ratio was approximately 1:7, so the combination picture was as realistic as possible and close to the real proportions of the human body. An example is shown in Figure 1.

The same faces and bodies were presented separately as the control stimuli. The stimulus presented alone remained as the composite stimulus with the same size and position on the screen. Only the outer outline of the head and the gray background of the face are “filled in” bodily expressions. Facial stimulation covered the head only.

### Design

A 2 (facial expressions: positive, negative) × 2 (bodily expressions: positive, negative) × electrode points (P1: O1, Oz, and O2; N170: P7, P8, PO7, PO8; N2: F1, FZ, and F2; P3: PZ, P1, and P2) design was selected. The dependent variables were the peak, latency, and average amplitude of the ERP components.

When isolated facial and bodily expressions were presented, the experimental design was 2 (stimulus: face, body) × 2 (expressions: positive, negative).

### Procedure

This is a two-part experiment: the consistency experiment, which presents the face–body compound stimuli, and the control experiment, in which the same participants see the face and body separately. Each part included two blocks, and all stimuli were randomly repeated twice in each block. Thus, each block had 120 trials, and each part had 240 trials, for a total of 480 trials. The order of pressing keys was counterbalanced among participants, and the order of the blocks was random.

**FIGURE 2:**
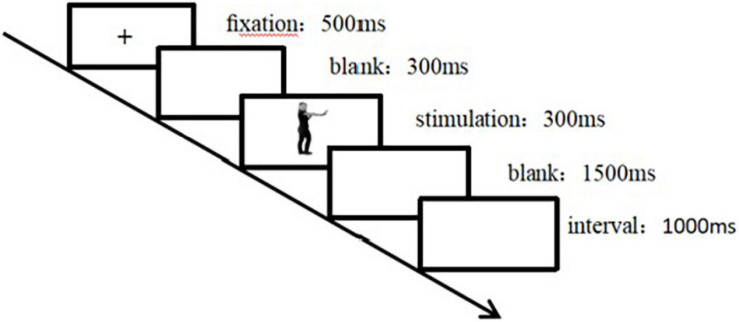
Flowchart of experimental trial procedure. Each trial started with a 500-ms fixation cross “+,” slightly above the center of the screen, which is the “chest height” of the body in the picture. After 300 ms of a blank screen, 300 ms of target stimulation was presented, followed by 1,500 ms of a blank screen. If there was no response within 1,500 ms, the blank screen disappeared automatically. After a 1,000-ms interval, the next trial started.

The experiment was conducted in an electromagnetically shielded and quiet electroencephalographic (EEG) laboratory. Participants sat comfortably in a chair with their eyes approximately 80 cm away from the screen. The size of the face–body compound stimulus presented on the screen was approximately 4 × 8 cm. The stimulus included isolated bodily expressions, isolated facial expressions, and compound expressions. The participants pressed “*F*” for positive and “J” for negative, and the left and right buttons were counterbalanced among participants. Figure 2 shows the flowchart of the trial procedure. Each trial started with a 500-ms fixation cross “+,” slightly above the center of the screen, which is the “chest height” of the body in the picture. Then, an empty screen was presented for 300 ms, the stimulus (isolated body expression/isolated facial expression/compound expression) was presented for 300 ms, and then an empty screen was presented for 1,500 ms. After the participant responded, the stimulus disappeared. If there was no response within 1,500 ms, the blank screen disappeared automatically and the next trial began after a 1,000-ms interval. In the compound presentation, participants were asked to judge the facial expressions in half of the trials and the bodily expressions in the remaining half. In the isolated presentation, the participants were asked to judge the expressions conveyed by the face or the body. This study utilized a block design, in which the block was a random factor.

### Data Recording

The EEG was recorded from a 64-channel Ag/AgCl electrode cap of the international 10–20 system. A vertical electrooculogram was recorded above and below the left eye, and a horizontal electrooculogram was positioned 10 mm from the outer canthus of each eye. During the online recording, the central forehead electrode was grounded, and the overhead electrode was used as the reference electrode. Signals were sampled at 1,000 Hz using AC mode sampling. The impedance was maintained below 5KΩ.

### Data Analysis

Curry 7 software was used for the offline analysis. All EEG signals were re-referenced to the average of the entire head. The EEG data were segmented into periods of 1,000 ms, including a 200-ms pre-stimulus baseline. Offline correction of eye movement artifacts was performed. Trials with voltages exceeding ± 100 μV at any electrode were discarded to exclude artifacts. After artifact rejection, no more than 20% of the trials were excluded. For isolated positive and negative face and body, and compound stimuli, about 48–56 trials were retained. A zero-phase waveform was selected for filtering, low-pass 30 Hz, and behavioral data were fused for segmentation and superposition.

P1, N170, N2, and P3 were analyzed according to the experimental purpose, group average waveform, and related literature (Meeren et al., 2005; Gu et al., 2013). P1 (60–140 ms) occipital sites were O1, Oz, and O2. N170 (120–200 ms) occipito-temporal sites were P7, P8, PO7, and PO8. N2 (200–300 ms) sites were F1, FZ, and F2. P3 (200–650 ms) sites were PZ, P1, and P2. The peak amplitudes and latencies of P1, N170, and N2 were analyzed, and the average amplitude of P3 was analyzed. The isolated and compound stimuli, P1 and N170, were analyzed, and for N2 and P3, only face and body compound presentation was analyzed. Repeated-measures ANOVA was employed to analyze the amplitude/latency of P1 and N170. For N2 and P3, the positive-face with positive-body and negative-face with negative-body were combined into one level (consistent), while the positive-face with negative-body and negative-face with positive-body were combined into one level (inconsistent), and the data were analyzed by one-way ANOVA.

## Results

### Behavioral Results

When presented with isolated faces and bodies, the main effect of stimulus was significant on accuracy [*F*(1, 27) = 17.432, *p* < 0.001, η*^2^* = 0.395] and RTs [*F*(1, 27) = 12.433, *p* < 0.01, η*^2^* = 0.321]. The RTs and accuracy for the face were faster and higher, respectively, than those for the body (606.142 ± 103.023 ms vs. 661.563 ± 97.296 ms; 87.322 ± 8.633 vs. 81.399 ± 8.578). The values reported in brackets are mean ± SD (*M* ± SD).

When judging facial expressions for the compound stimuli, the interaction between facial and bodily expressions was significant for both accuracy [*F*(1, 27) = 14.302, *p* < 0.01, η*^2^* = 0.357] and RTs [*F*(1, 27) = 19.481, *p* < 0.001, η*^2^* = 0.422]. Simple-effect analysis showed that when the faces were accompanied by bodies with congruent expressions relative to incongruent expressions [*F*(1, 27) = 10.309, *p* < 0.01, η*^2^* = 0.297], participants made significantly faster (655.431 ± 120.158 ms vs. 633.021 ± 103.750 ms) and better (88.731 ± 7.963 vs. 83.021 ± 8.381) decisions. When judging bodily expressions for the compound stimuli, the interaction between facial and bodily expressions was significant for both accuracy [*F*(1, 27) = 17.792, *p* < 0.001, η*^2^* = 0.412] and RTs [*F*(1, 27) = 21.312, *p* < 0.001, η*^2^* = 0.448]. Simple-effect analysis showed that when the bodies were accompanied by faces with congruent expressions relative to incongruent expressions [*F*(1, 27) = 13.661, *p* < 0.01, η*^2^* = 0.342], participants made significantly faster (613.226 ± 98.430 ms vs. 649.106 ± 102.619 ms) and better (87.837 ± 8.364 vs. 81.264 ± 7.981) decisions.

### Event-Related Potential Results

#### P1

When presented with isolated faces and bodies, the main effect of stimulus was significant on the peak amplitude [*F*(1, 27) = 5.904, *p* < 0.05, η*^2^* = 0.179]. The P1 amplitude was larger for the body (5.034 ± 2.790 μV) than for the face (4.000 ± 2.656 μV). The interaction between stimulus and emotion was significant [*F*(1, 27) = 8.402, *p* < 0.01, η*^2^* = 0.237]. Simple-effect analysis showed that the amplitude was larger for a negative body than for a negative face (5.284 ± 2.758 μV vs. 3.737 ± 2.312 μV) [*F*(1, 27) = 11.93, *p* < 0.01, η*^2^* = 0.305].

When presented with isolated faces and bodies, the main effect of stimulus was significant on the latency [*F*(1, 27) = 16.952, *p* < 0.001, η*^2^* = 0.386]. The P1 latency of the face was longer than that of the body (114.012 ± 21.996 ms vs. 100.952 ± 18.660 ms).

When judging facial expressions for the compound stimuli, only the main effect of electrode points was significant on the peak amplitude [*F*(2, 26) = 4.580, *p* < 0.05, η*^2^* = 0.261], with O2 > O1 > OZ. The results of *post hoc* testing showed that O2 (4.915 ± 2.192μV) had significantly higher peak amplitude than OZ (4.479 ± 2.181 μV; *p* < 0.05). When judging bodily expressions for the compound stimuli, no other effects were found.

#### N170

When presented with isolated faces and bodies, the main effect of stimulus was significant on the peak amplitude [*F*(1, 27) = 20.394, *p* < 0.001, η*^2^* = 0.43]. The N170 amplitude was larger for the face than for the body (−5.891 ± 3.761 μV vs. −4.360 ± 3.976 μV). A main effect of emotion was significant on the peak amplitude [*F*(1, 27) = 7.040, *p* < 0.05, η*^2^* = 0.207], showing larger amplitude for positive than for negative (−5.338 ± 4.00 μV vs. −4.913 ± 3.734 μV). The interaction between stimulus and emotion was significant for the peak amplitude [*F*(1, 27) = 16.683, *p* < 0.001, η*^2^* = 0.382]. Simple-effect analysis showed that the amplitude was larger for a positive body compared with a negative body (−4.86 ± 4.160 μV vs. −3.861 ± 3.791 μV) [*F*(1, 27) = 23.97, *p* < 0.001, η*^2^* = 0.47].

When presented with isolated faces and bodies, the main effect of stimulus was significant on the latency [*F*(1, 27) = 34.224, *p* < 0.001, η*^2^* = 0.559]. The N170 latency of the face was longer than that of the body (170.987 ± 11.874 ms vs. 159.915 ± 16.674 ms). The interaction between stimulus and emotion was significant for the latency [*F*(1, 27) = 4.374, *p* < 0.05, η*^2^* = 0.139]. Simple-effect analysis showed that the latency of face was longer than that of the body whether emotion is positive [*F*(1, 27) = 21.45, *p* < 0.001, η*^2^* = 0.443] or negative [*F*(1, 27) = 36.30, *p* < 0.001, η*^2^* = 0.573].

When judging facial expressions for the compound stimuli, only the main effect of electrode points was significant on the peak amplitude [*F*(3, 25) = 7.699, *p* < 0.01, η*^2^* = 0.48], with P8 > P7 > PO8 > PO7. The results of *post hoc* testing showed that P7 (−5.880 ± 3.971 μV) and P8 (−6.192 ± 3.782 μV) were significantly higher in terms of peak amplitude than PO7 (−4.465 ± 3.84 μV; *p* < 0.001, *p* < 0.05, respectively). When judging bodily expressions for the compound stimuli, a main effect of the electrode points was significant on the peak amplitude [*F*(3, 22) = 5.922, *p* < 0.01, η*^2^* = 0.447], with P8 > PO8 > P7 > PO7. The results of *post hoc* testing showed that P8 (−6.190 ± 4.319 μV), PO8 (−6.009 ± 5.412 μV), and P7 (−5.238 ± 3.777 μV) had significantly higher peak amplitude than PO7 (−4.121 ± 3.625 μV; *p* < 0.01).

When judging facial expressions for the compound stimuli, only the main effect of electrode points was significant on the latency [*F*(3, 25) = 4.150, *p* < 0.05, η*^2^* = 0.332], with P7 > PO7 > P8 > PO8. The results of *post hoc* testing showed that P7 (164.518 ± 11.782 ms), PO7 (161.795 ± 13.732 ms), and P8 (159.214 ± 10.657 ms) had significantly longer latency than PO8 (152.795 ± 13.127 ms; *p* < 0.01), and P7 had significantly longer latency than P8 (*p* < 0.05). When judging bodily expressions for the compound stimuli, the main effect of the electrode points was significant on the latency [*F*(3, 22) = 4.948, *p* < 0.01, η*^2^* = 0.403], with P7 > PO7 > P8 > PO8. The results of *post hoc* testing showed that P7 (163.900 ± 12.730 ms), PO7 (162.103 ± 11.982 ms), and P8 (160.220 ± 9.887 ms) had significantly longer latency than PO8 (155.310 ± 11.366 ms; *p* < 0.01). The interaction between bodily expressions and electrode points was significant for latency [*F*(3, 22) = 8.635, *p* < 0.01, η*^2^* = 0.541]. The interaction between facial and bodily expressions was significant [*F*(1, 24) = 10.442, *p* < 0.01, η*^2^* = 0.303]. Simple-effect analysis showed that negative face–negative body was longer than positive face–negative body (162.38 ± 15.806 ms vs. 159.14 ± 18.242 ms) [*F*(1, 24) = 4.521, *p* < 0.05, η*^2^* = 0.159].

#### N2

When judging facial and bodily expressions for the compound stimuli, the main effect of consistency was significant on the peak amplitude [*F*(1, 23) = 6.72, *p* < 0.05, η*^2^* = 0.530], showing a larger amplitude for inconsistency than for consistency (−4.02 ± 3.793 μV vs. −3.969 ± 3.564 μV).

When judging facial and bodily expressions for the compound stimuli, the main effect of consistency was significant on the latency [*F*(1, 23) = 16.570, *p* < 0.01, η*^2^* = 0.472], showing a shorter latency for consistency than for inconsistency (254.277 ± 36.372ms vs. 265.780 ± 30.222ms).

#### P3

**FIGURE 3:**
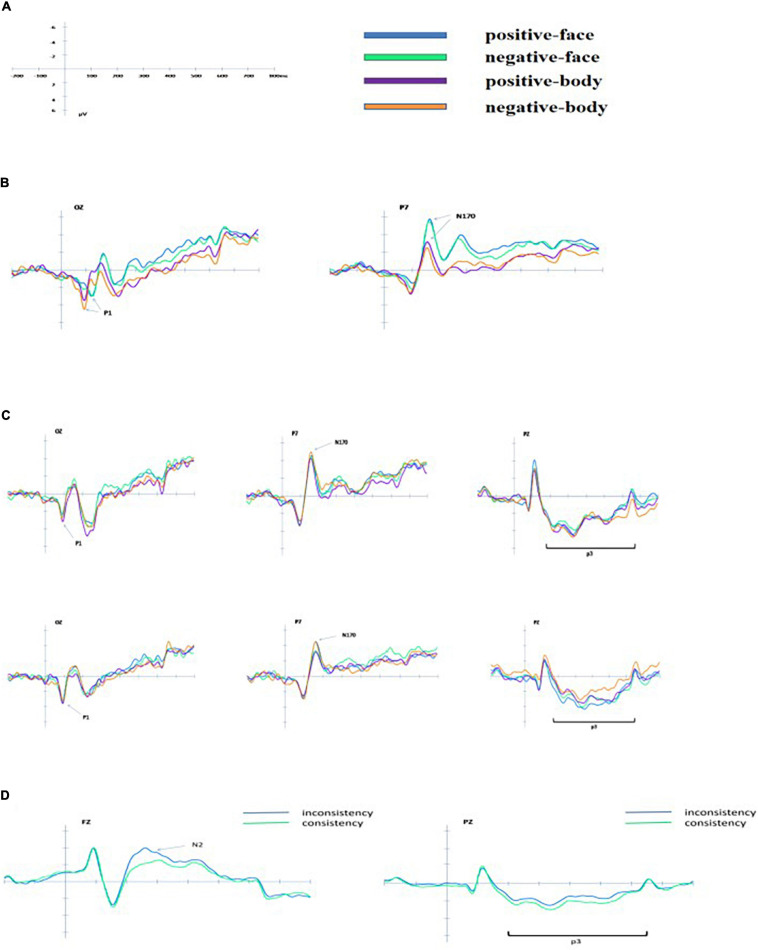
The grand average ERPs for each condition. **(A)** Schematic diagram of the scale legend. **(B)** Group average waveforms of electrode for isolated positive-face, negative-face, and positive-body, negative-body on OZ and P7 (only partial electrode points are shown). **(C)** Group average waveforms of electrode for compound facial and bodily expressions on OZ, P7, and PZ (upper: judging the face; bottom: judging the body). **(D)** Group average waveforms of the electrode for compound facial and bodily expressions (consistency and inconsistency) on FZ and PZ (left: N2 on FZ; right: P3 on PZ).

When judging facial expressions for the compound stimuli, no significant effect was found; when judging bodily expressions for the compound stimuli, the interaction between facial and bodily expressions was significant [*F*(1, 24) = 10.536, *p* < 0.01, η*^2^* = 0.305]. The main effect of consistency was significant [*F*(1, 23) = 14.113, *p* < 0.01, η*^2^* = 0.380], showing greater amplitude for consistency than for inconsistency (2.882 ± 2.446 μV vs. 2.287 ± 2.459 μV). Figure 3 shows the ERP data for each condition.

## Discussion

### P1: Body Received Attention First and There Was Stimulus Effect

The results showed that the main effect of a stimulus was significant. For P1, the peak amplitude of the body was larger but the latency was shorter, which is inconsistent with the results of Meeren et al. (2005). In our study, positive and negative facial and bodily expressions were used, while Meeren et al. used negative emotions (fear and anger). However, significant differences were found only for the presentation of isolated stimuli and not for compound stimuli, as in Meeren et al. It is believed that P1, as an exogenous component of early perceptual processing, is sensitive to low-level threat information of stimulation. However, bodily expressions are lower-frequency information than facial expressions, thus inducing a greater P1 component (Pourtois et al., 2005). The shorter latency of the body indicated that the body received attention before the face, which is consistent with the findings of Gu et al. (2013). However, the inconsistency with Gu et al. (2013) was that P1 had no bias to negative emotions. A possible reason is that the body is larger than the face, so the participant pays attention to the body first. Therefore, the proportion of face–body size in this study is worthy of attention. This study did not find a bias of P1 toward negative emotions, which may be due to the different materials selected in this experiment. The negative emotions selected in this study included anger, disgust, fear, sadness, and surprise, while the positive emotions contained only one expression (happiness). Thus, the group of positive expressions is more perceptually homogenous, as they depict only a single expression, while the negative expressions are more heterogeneous, as they depict several emotional expressions. Indeed, there is a possibility that different emotions in compound stimuli could have different effects (Karaaslan et al., 2020). Therefore, further research is required. Although the main effect of expression was not found, the amplitude of the negative body was larger than that of the negative face, indicating that the P1 component was affected by negative emotion under the influence of stimulation. In addition, our research conducted using different tasks found no difference except for the electrode.

### N170: At the Stage of Structure Coding, Interaction Begins to Exist

The main effect of a stimulus was significant, the peak amplitude of the face was larger, and the latency was longer. N170 reflects the processing of configuration, and many studies have found N170 is induced by the face (Zhang et al., 2015). The body also triggered N170, but the amplitude was not as large as that of the face. To a certain extent, facial and bodily expressions have the same processing characteristics (Aviezer et al., 2012b; Kret et al., 2013; Poyo Solanas et al., 2018). The results suggest that, from P1 to N170, facial emotions began to play a role. The main effect of emotion was significant, and the positive peak amplitude was greater than the negative peak amplitude, indicating that emotion was further processed at this stage. Therefore, at this stage, we not only distinguish the stimulus but also distinguish emotional valence. This may be because facial expressions are the primary way to judge emotion in daily life and positive emotions attract more attention. However, some studies have suggested that N170 does not reflect emotion, while others have found that it has an emotional effect (Eimer and Holmes, 2002; Batty and Taylor, 2003; Hietanen and Nummenmaa, 2011; Schindler et al., 2021). The main effect of bodily expressions was significant, and the positive effect was greater than the negative effect. This indicates that bodily expressions were processed at this time, and some studies have found that the effect of bodily expressions was found earlier (Gu et al., 2013).

When judging facial expressions for the compound stimuli, the body had little effect on the face, and there was no significant effect. However, the face advantage of N170 did not exist in comparison with the isolated presentation. This indicates that the influence of the body on the face may have occurred at a relatively early stage; when judging bodily expressions, the effect began to appear. The interaction between facial and bodily expressions was significant, and the consistent latency was longer than the inconsistent latency (Syrjänen et al., 2018). The N170 of compound stimulation seemed sensitive to both the inconsistency between facial and bodily expressions and the stimulus. A possible reason is that individuals have completed the preliminary judgment of the body in the early stage and then turned to the structural coding of the face. In this stage, they can process the face and body simultaneously. The different tasks of the experiment led to different results. For the compound stimuli, the faces and bodies were fairly clearly photoshopped together, which may have disrupted the perception of participants of them as a single stimulus.

### N2 and P3: Further Processing of Conflict Emotion Information and Evaluation

The main effect of consistency on amplitude and latency was significant, showing greater amplitude and longer latency for inconsistency than for consistency.

This finding indicates that the emotional conflict information was sensitive and lasted for a long time. The interaction between facial and bodily expressions was significant at P3. The average amplitude was greater for consistency than for inconsistency, indicating continuous attention to consistent emotional information. Participants may have first paid attention to inconsistent information because it is more prominent than consistent information and then classified and evaluated emotional stimuli using high-level cognitive processing.

## Conclusion

The findings of this study are as follows. (1) There is an interaction between facial and bodily expressions. (2) In the early stage (P1), participants focus first on bodily expressions (stimulus effect), but facial expressions rely more on emotional judgment, and they are dominant in the middle stage (N170). Finally, higher-level processing of inconsistencies and consistencies between emotions could distinguish between different emotions, and emotional conflict was emphasized. Overall, the findings support the three stages of processing facial and bodily expressions, but the characteristics of the specific stages differ (Luo et al., 2010; Gu et al., 2013). (3) The processing of facial and bodily expressions differ; the interaction between facial and bodily expressions occurred in the early stage, the characteristics of interaction were not completely consistent, and the influence of the face on the body was long-lasting.

## Data Availability Statement

The datasets presented in this study can be found in online repositories. The names of the repository/repositories and accession number(s) can be found below: https://figshare.com/articles/dataset/Psychological_behavior_data_and_ERP_data/14215889 DOI: 10.6084/m9.figshare.14215889.

## Ethics Statement

Ethical review and approval was not required for the study on human participants in accordance with the local legislation and institutional requirements. All participants gave written informed consent in accordance with the 2013 Declaration of Helsinki and were paid for their attendance.

## Author Contributions

XL designed and performed the experiment, performed the statistical analysis, wrote the manuscript, and approved the final version of the manuscript for submission.

## Conflict of Interest

The author declares that the research was conducted in the absence of any commercial or financial relationships that could be construed as a potential conflict of interest.
